# Acute Effects of Small-Sided Games and Tabata High-Intensity Interval Training on Physical, Psychophysiological, and Cognitive Responses in Male Soccer Players

**DOI:** 10.3390/life16040646

**Published:** 2026-04-11

**Authors:** Alirıza Han Civan, Adem Civan, Mahmut Esat Uzun, Soner Akgün, Enes Akdemir, Ali Kerim Yılmaz

**Affiliations:** 1 Faculty of Hasan Doğan Sports Sciences, Karabük University, Karabük 78000, Türkiye; alirizahancivan@karabuk.edu.tr (A.H.C.); mahmutuzun@karabuk.edu.tr (M.E.U.); 2 Faculty of Sports Sciences, Selçuk University, Konya 42250, Türkiye; acivan@selcuk.edu.tr; 3 Faculty of Sports Sciences, Artvin Çoruh University, Artvin 08100, Türkiye; 4 Faculty of Yaşar Doğu Sports Sciences, Ondokuz Mayıs University, Samsun 55270, Türkiye; akdemrenes@gmail.com (E.A.); akerim.yilmaz@omu.edu.tr (A.K.Y.)

**Keywords:** small-sided games, high-intensity interval training, neuromuscular fatigue, cognitive performance, soccer

## Abstract

Background: Small-sided games (SSG) and running-based high-intensity interval training (HIIT) are commonly used in soccer conditioning to improve aerobic fitness and performance. Although both modalities induce high cardiovascular stress, their acute neuromuscular, perceptual, and cognitive responses remain incompletely understood when examined within the same cohort. This study compared the acute physical, psychophysiological, and cognitive responses to SSG and Tabata-type HIIT in amateur male soccer players. Methods: Thirty-two male amateur players (*n* = 32; age: 20.53 ± 1.65 years) completed a counterbalanced within-subject crossover design. Participants performed a 4v4 SSG protocol and a running-based Tabata-HIIT protocol (8 × 20 s, 10 s recovery) on separate days (48 h apart). Countermovement jump (CMJ), squat jump (SJ), 20-m sprint, agility *t*-test, heart rate, perceived exertion (Borg CR-10), mental effort, and cognitive performance (d2 test) were assessed pre- and post-exercise. Parametric variables were analyzed using 2 × 2 repeated-measures ANOVA (time × protocol; η^2^p), and non-parametric data were analyzed using Friedman and Wilcoxon tests (r) (*p* < 0.05). Results: Both protocols elicited similar cardiovascular responses (~90% HR_max_). A significant protocol × time interaction was observed for CMJ (*p* < 0.001), showing a decline after Tabata-HIIT, whereas performance was maintained after SSG. No inter-protocol differences were found for SJ, sprint, or agility. Perceived exertion and mental effort during recovery were higher following Tabata-HIIT (*p* < 0.05). Cognitive performance improved after both protocols (*p* < 0.001), with no between-protocol differences. Conclusions: Despite comparable cardiovascular load, Tabata-HIIT was associated with greater acute neuromuscular and perceptual strain, whereas SSG preserved neuromuscular performance. Perceptual and mental responses may therefore differ despite similar physiological intensity, which may inform soccer training prescription.

## 1. Introduction

Optimizing athletic performance in soccer is largely dependent on the level of representation in training of the physiological and mechanical demands that arise during matches [1]. This perspective is based on the principle of specificity, which aims to optimize performance transfer by increasing the alignment of training stimuli with competition conditions. Soccer is characterized by high-intensity running, short repeated sprints, and sudden changes of direction. Furthermore, it is critical that players continuously process sensory information and make correct decisions within a limited time [2]. Therefore, contemporary training methods applied in soccer should not only create physiological loads consistent with the competitive situation, but also adequately reflect perceptual and cognitive complexity.

High-intensity interval training (HIIT) is widely used in soccer-specific conditioning due to its ability to elicit substantial cardiovascular and metabolic responses. Recent evidence indicates that HIIT can effectively improve both aerobic and anaerobic performance parameters in soccer players [3,4]. For this reason, the Tabata protocol which integrates 10-s rest intervals with 20-s periods of maximum effort has garnered particular attention due to its ability to generate a significant physiological load within a relatively short training duration [5,6]. However, some studies also indicate that running-based high-intensity training protocols can cause significant fatigue, metabolic stress, and a partial decrease in explosive power in the short term [7,8].

Small-sided games (SSG) are a game-based training method designed to reflect the specific requirements of soccer. SSG formats played on different field sizes and with varying numbers of players not only stimulate perceptual scanning, rapid decision-making, and technical–tactical interactions but also generate a significant level of physiological stress [9,10]. Research indicates that when field dimensions and player numbers are appropriately adjusted, SSG provides exercise intensities corresponding to approximately 85–95% of maximum heart rate (HR_max_) and generates cardiovascular loads similar to those observed in running-based HIIT protocols [11,12]. Additionally, match analysis results show that players’ individual HR_max_ vary between 80% and 90% during competitive situations [13].

Recent studies have found that SSG and HIIT methods induce similar levels of physiological stress in soccer players [14,15,16,17]. However, although no significant difference in perceived exertion was observed between SSG and HIIT methods [14,16], it is frequently noted that SSG is rated as more enjoyable by soccer players compared to running-based HIIT protocols [17]. These findings suggest that game-based training protocols may provide important benefits regarding participant tolerability and the sustainability of exercise programs. Furthermore, most similar studies in literature have focused on the chronic adaptations of SSG and HIIT methods, while the acute neuromuscular, perceptual, and cognitive effects of these methods have been examined to a limited extent [18,19]. However, examining the acute effects of similar cardiovascular training on fatigue development, perceptual load, and cognitive changes is critical for performance optimization [20,21]. On the other hand, while some studies have found that exercise temporarily enhances cognitive functions such as attention, processing speed, and concentration [22,23], it is observed that these studies were mostly conducted in laboratory settings, and there is a limited number of studies investigating the physical, psychophysiological, and cognitive responses of soccer-specific training models [24,25,26]. In this context, the extent to which SSG and running-based HIIT protocols differ in terms of their immediate physical, perceptual, and cognitive effects has not yet been sufficiently clarified. Therefore, the present study has the potential to fill this significant gap in literature.

In light of this information, the aim of this study is to compare the acute physical, psychophysiological, and cognitive responses of amateur soccer players following participation in SSG and Tabata-HIIT. The other aim of this study is not to directly compare the exercise loads of the SSG and Tabata-HIIT protocols, but rather to evaluate the acute responses of these two training approaches, which are widely used in practice and have structurally distinct characteristics. This study hypothesized that SSG and Tabata-HIIT would elicit similar internal load responses but produce different acute effects on physical and cognitive performance outcomes.

## 2. Materials and Methods

### 2.1. Experimental Design

**Figure 1:**
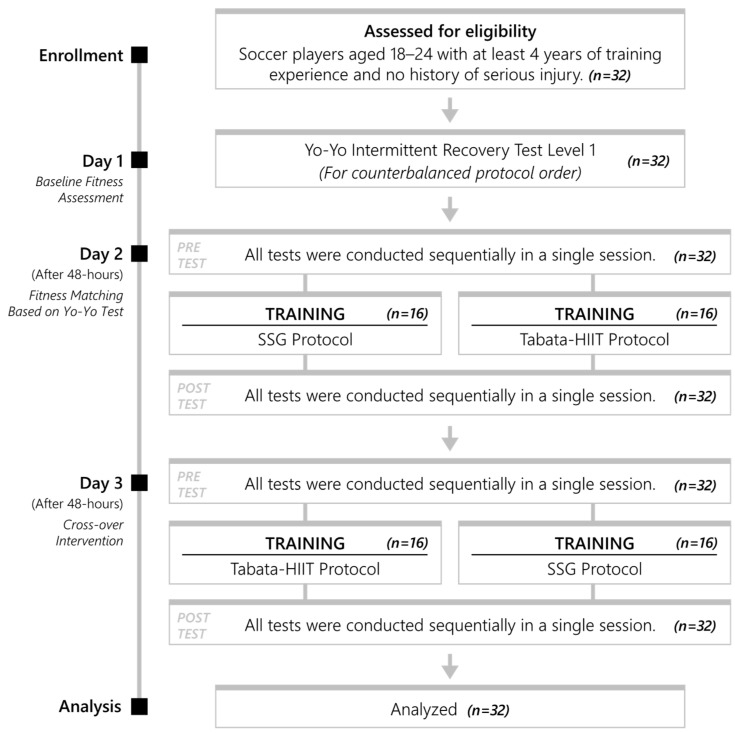
Flowchart.

The study included 32 male soccer players who actively play amateur soccer and volunteered to participate. A counterbalanced within-subject cross-over design was used to examine and compare the acute effects of SSG and Tabata-style HIIT on the physical, psychophysiological, and cognitive responses of soccer players. To control for potential variability in aerobic fitness, participants were stratified according to their performance on the Yo-Yo Intermittent Recovery Test Level 1 and subsequently allocated to the exercise conditions in a counterbalanced sequence (Figure 1). Participants were divided into two equal groups (*n* = 16 per group). Participants were divided into two groups to ensure a homogeneous distribution based on their initial aerobic performance levels (as determined by Yo-Yo Intermittent Recovery Test results). Subsequently, within a counterbalanced crossover design, both exercise protocols were administered in different sequences (Group 1: SSG → Tabata-HIIT; Group 2: Tabata-HIIT → SSG). This approach aims to counteract potential order effects associated with the sequence of administration [27]. On Day 1, both groups completed baseline assessments, including anthropometric measurements and the Yo-Yo test. On Day 2, Group 1 performed the SSG protocol, whereas Group 2 completed the Tabata-HIIT protocol. On Day 3, the exercise conditions were reversed, with Group 1 performing Tabata-HIIT and Group 2 completing SSG. This counterbalanced crossover structure ensured that all participants were exposed to both exercise modalities in different sequences, thereby minimizing potential order effects. A 48-h recovery interval was implemented between experimental sessions to reduce residual fatigue. The 48-h recovery period implemented between protocols is designed to mitigate the effects of acute fatigue and is a practice commonly adopted in high-intensity exercise studies [20]. However, given the delayed recovery processes that may occur, particularly in the neuromuscular and cognitive systems, it is not possible to assume that this period completely eliminates all residual effects [28]. The SSG and Tabata-style HIIT protocols used in this study differ in terms of duration, the temporal distribution of exercise intensity, movement characteristics, and work-to-rest ratios. These differences were preserved in the study design to maintain the unique application characteristics of both training models. Accordingly, each protocol was evaluated within its own characteristic structure. Familiarization sessions for the performance tests were conducted, and participants were introduced to the procedures of the tests.

Written informed consent was obtained from all participants. The study protocol conformed to the principles outlined in the Declaration of Helsinki and received approval from the Karabük University Non-Interventional Clinical Research Ethics Committee (Decision No: 2025/2504).

### 2.2. Participants

The study included 32 male amateur soccer players aged 18–24 who had been competing as licensed players for at least four years and trained at least three days a week. The descriptive information of the participants is presented in Table 1. An additional inclusion criterion was that participants had not experienced any serious musculoskeletal injuries in the past six months and had signed a written informed consent form. Exclusion criteria comprised individuals outside the specified age range; those with chronic medical conditions or regular medication use; those presenting with neurological, orthopedic, or cardiovascular disorders; those with any health-related limitation that could interfere with consistent participation; and individuals who declined to sign the informed consent form.

G*Power analysis (version 3.1) was conducted to determine the sample size. Based on an assumed moderate effect size (f = 0.25), 80% statistical power, and a 0.05 alpha level, the required minimum sample size was calculated to be 28 participants. Taking into account potential participant or data loss during the process, the number of volunteers was set at 32.

### 2.3. Experimental Procedure

All assessments were conducted at a standardized time of day (±1 h) under comparable environmental conditions to limit circadian and contextual variability. Upon arrival, participants completed the d2 attention test in a quiet setting prior to the warm-up to prevent acute physical fatigue from influencing baseline cognitive performance. A standardized warm-up protocol lasting approximately 10–12 min was then performed, consisting of low-intensity running, dynamic stretching exercises, and short accelerations. Following the warm-up, participants were equipped with Polar heart rate monitors, and heart rate data were continuously recorded throughout the training sessions. Pre-intervention physical performance tests were administered in a consistent order across all sessions: Countermovement Jump (CMJ), Squat Jump (SJ), 20-m sprint, and the agility *t*-test. After completion of the pre-tests, participants performed the designated training protocol (SSG or Tabata-HIIT). Immediately following the intervention, the same battery of physical performance tests (CMJ, SJ, 20-m sprint, and *t*-test) was repeated, along with re-administration of the d2 attention test. Each session concluded with an approximately 5-min cool-down period comprising low-intensity running and stretching exercises. Ratings of perceived exertion (RPE) and mental effort were recorded immediately after each exercise set and again 10 min following the completion of the training protocol. Participants were instructed to maintain their nutrition, hydration, and sleep patterns and to avoid strenuous physical activity prior to the test sessions.

### 2.4. Measurements

#### 2.4.1. Anthropometric Measurements

Participants’ stature was assessed using a wall-mounted stadiometer with a measurement precision of 0.01 cm (Holtain Ltd., Crymych, UK). Body mass and body fat percentage were determined using bioelectrical impedance analysis with an InBody 270 body composition analyzer (InBody Co., Ltd., Seoul, Republic of Korea).

#### 2.4.2. Yo-Yo Interval Recovery Test (Level 1)

Aerobic capacity was evaluated using the Yo-Yo Intermittent Recovery Test Level 1. The test was administered on a natural grass surface in accordance with the standardized protocol described by Bangsbo et al. [29].

#### 2.4.3. Heart Rate Measurement

Physiological load during the training sessions was monitored using the Polar Team system. Throughout each exercise protocol, mean heart rate (HR_mean_), peak heart rate (HR_peak_), and percentage of maximal heart rate (%HR_max_) were continuously recorded and subsequently analyzed to quantify the cardiovascular demands imposed by the training interventions. HR_max_ was determined using the age-based prediction equation (HR_max_ = 220 − age), which is widely used in field-based exercise studies. This approach is considered a valid method for monitoring exercise intensity under field conditions where the practical application of direct maximal tests is limited [20]. The %HR_max_ values for the participants were calculated based on these individual estimated maximum values.

#### 2.4.4. Countermovement Jump (CMJ)

Vertical jump performance was assessed using the CMJ test. Measurements were obtained with a Smartspeed jump mat system (Fusion Sport, Queensland, Australia). Participants performed the jumps with their hands positioned on their hips to eliminate arm swing contribution. Two trials were completed, and the highest jump height was retained for analysis [30].

#### 2.4.5. Squat Jump (SJ)

The SJ test was conducted using the same measurement system as the CMJ. Participants assumed a fixed starting position with approximately 90° of knee flexion and executed a maximal vertical jump without any preparatory countermovement. The test was administered twice within each session (pre- and post-protocol), with two trials performed at each time point. The best performance was included in the statistical analysis [30].

#### 2.4.6. Sprint Test

Linear sprint performance was evaluated using a 20-m sprint test. Sprint times were recorded with the Smartspeed electronic photocell timing system (Fusion Sport, Queensland, Australia). Each participant performed two maximal attempts, and the fastest time was used for subsequent analyses [31].

#### 2.4.7. Agility Test (*t*-Test)

Change-of-direction ability was assessed using the *t*-test protocol. Timing was measured with the Smartspeed gate system. Two trials were conducted, and the best recorded time was included in the analysis [32].

#### 2.4.8. Cognitive Performance—D2 Attention Test

Cognitive functioning was assessed using the d2 Attention Test. This standardized paper-and-pencil neuropsychological instrument evaluates selective attention, processing speed, and concentration capacity [33]. For statistical analyses, Total Number of Items Processed (TN), Total Errors, and Concentration Performance (CP) scores were considered [34].

#### 2.4.9. Perceived Effort Scale

The perceived level of difficulty was assessed using the Borg CR-10 Perceived Effort Scale [35]. Participants were asked to report their perceived level of difficulty throughout the session at the end of each set and 10 min after the completion of the training [11].

#### 2.4.10. Mental Effort (ME)

Mental effort was evaluated using the Mental Effort Scale developed by Zijlstra [36]. This scale consists of a vertical continuum ranging from 0 (“absolutely no effort”) to 150 (“extreme effort”). Assessments were conducted concurrently with the Borg CR-10 ratings, immediately after each set and 10 min post-exercise, to provide an integrated evaluation of the overall cognitive strain experienced during the session.

#### 2.4.11. Small-Sided Games (SSG)

The SSG protocol was conducted in a 4 vs. 4 format on a 25 m × 35 m artificial turf pitch. The session consisted of four 4-min bouts, interspersed with 2 min of passive recovery. Players received only verbal encouragement during play; no tactical or technical instructions were provided. Additional balls were positioned around the field to maintain continuous game flow and minimize interruptions [37,38,39].

#### 2.4.12. Tabata-HIIT Protocol

The Tabata-style HIIT intervention followed a 20-s maximal effort and 10-s passive recovery structure. Participants performed repeated 10–20 m shuttle runs incorporating a 180° change of direction. The protocol consisted of a single set of eight repetitions [5].

### 2.5. Data Analysis

All statistical analyses were performed using IBM SPSS Statistics for Windows (Version 26.0, IBM Corp., Armonk, NY, USA). Data are presented as mean ± standard deviation (SD). The normality of the data distribution was assessed using the Shapiro–Wilk test. Acute changes in physical performance and d2 attention variables were analyzed using a 2 × 2 repeated-measures ANOVA with time (pre vs. post) and protocol (SSG vs. Tabata-HIIT) as within-subject factors. The assumptions of repeated-measures ANOVA were assessed, and sphericity was confirmed using Mauchly’s test. Effect sizes were reported as partial eta squared (η^2^p). Heart rate variables (HR_mean_ HR_peak_, %HR_mean_, and %HR_max_) were analyzed using one-way repeated-measures analysis of variance to examine differences between SSG sets. For variables that did not meet normality assumptions (Borg CR-10 and mental effort), non-parametric tests were applied. Differences across SSG sets were examined using the Friedman test, while recovery responses (end of exercise vs. 10-min post) and inter-protocol comparisons were analyzed using the Wilcoxon signed-rank test. Effect sizes for non-parametric analyses were calculated as r (Z/√N). Heart rate responses during SSG were evaluated across sets using repeated-measures analysis. Statistical significance was set at *p* < 0.05.

## 3. Results

Detailed demographic and anthropometric characteristics of the participants are presented in Table 1. Thirty-two male amateur soccer players participated in the study. The participants had a mean age of 20.53 ± 1.65 years, height of 176.41 ± 3.83 cm, and body mass of 69.47 ± 7.34 kg. Mean skeletal muscle mass was 33.53 ± 3.63 kg, body fat percentage was 15.14 ± 4.87%, and training experience mean 6.53 ± 1.81 years.

**Figure 2:**
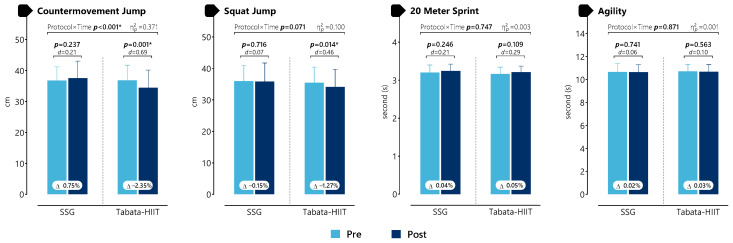
Acute Changes in Physical Performance Variables Following SSG and Tabata-HIIT Protocols. * *p* < 0.05.

As presented in Figure 2 and Table S1, a statistically significant protocol × time interaction was observed for CMJ (*p* < 0.001, η^2^p = 0.370), indicating a large effect size. No statistically significant interaction effects were identified for SJ (*p* = 0.071, η^2^p = 0.100), sprint (*p* = 0.747, η^2^p = 0.003), or agility (*p* = 0.871, η^2^p = 0.001), with effect sizes ranging from small to moderate. Within-condition analyses revealed statistically significant pre–post differences only for Tabata-HIIT in CMJ (*p* < 0.001, d = 0.69), indicating a moderate effect size, and SJ (*p* = 0.014, d = 0.46), indicating a moderate effect size. No other comparisons reached statistical significance (all *p* > 0.05), with effect sizes generally small.

**Figure 3:**
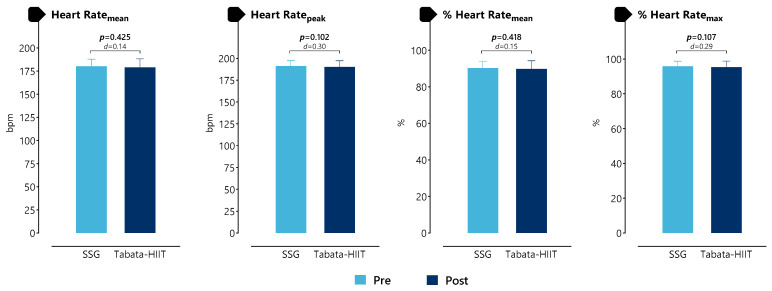
Comparison of mean heart rates in SSG and Tabata-HIIT protocols.

Figure 3 and Table S1 presents the results of a comparison of mean heart rates between the SSG and Tabata-HIIT protocols. No statistically significant differences were observed between protocols for HR_mean_ (*p* = 0.425, d = 0.14), HR_peak_ (*p* = 0.102, d = 0.30), %HR_mean_ (*p* = 0.418, d = 0.15), and %HR_max_ (*p* = 0.107, d = 0.29).

**Figure 4:**
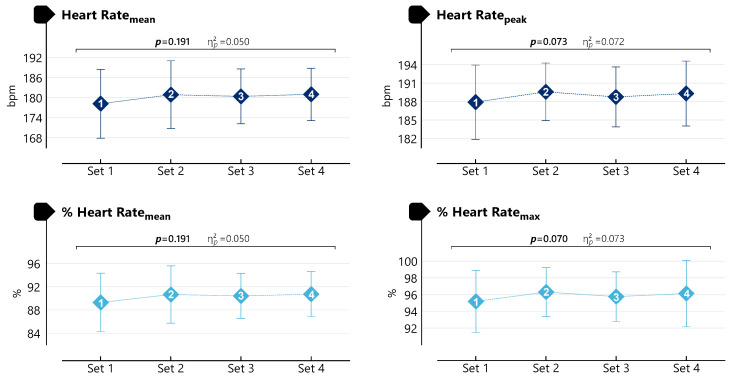
Heart rate responses during SSG.

As shown in Figure 4, no significant effect of set was observed for HR_mean_ (F(3,93) = 1.616, *p* = 0.191, η^2^p = 0.050), HR_peak_ (F(3,93) = 2.399, *p* = 0.073, η^2^p = 0.072), %HR_mean_ (F(3,93) = 1.614, *p* = 0.191, η^2^p = 0.050), and %HR_max_ (F(3,93) = 2.434, *p* = 0.070, η^2^p = 0.073).

**Figure 5:**
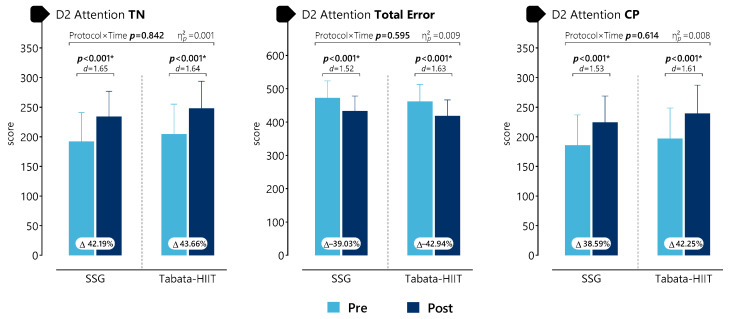
Acute effects of SSG and Tabata-HIIT on d2 attention outcomes. * *p* < 0.05.

The data presented in Figure 5 showed statistically significant pre–post differences for all d2 attention variables following both SSG and Tabata-HIIT. Significant increases were observed for TN in SSG (*p* < 0.001, d = 1.65) and Tabata-HIIT (*p* < 0.001, d = 1.64), and for CP in SSG (*p* < 0.001, d = 1.53) and Tabata-HIIT (*p* < 0.001, d = 1.61). A significant decrease was observed for Total Error in SSG (*p* < 0.001, d = 1.52) and Tabata-HIIT (*p* < 0.001, d = 1.63). No statistically significant protocol × time interaction effects were found for TN (F(1,31) = 0.04, *p* = 0.842, η^2^p = 0.001), Total Error (F(1,31) = 0.29, *p* = 0.595, η^2^p = 0.009), or CP (F(1,31) = 0.26, *p* = 0.614, η^2^p = 0.008).

**Figure 6:**
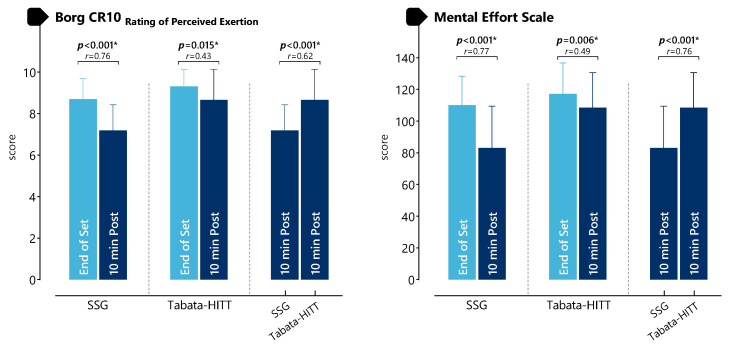
Recovery responses of perceived exertion and mental effort after SSG and Tabata-HIIT. * *p* < 0.05.

As presented in Figure 6 and Table S1, Borg CR-10 values significantly decreased from the end of exercise to 10 min post-exercise in both SSG (Z = −4.284, *p* < 0.001, r = 0.760) and Tabata-HIIT (Z = −2.422, *p* = 0.015, r = 0.430). At 10 min post-exercise, Borg values were significantly higher in Tabata-HIIT compared to SSG (Z = −3.499, *p* < 0.001, r = 0.620). Mental effort scores also significantly decreased from the end of exercise to 10 min post-exercise in both SSG (Z = −4.376, *p* < 0.001, r = 0.774) and Tabata-HIIT (Z = −2.762, *p* = 0.006, r = 0.490). At 10 min post-exercise, mental effort was significantly higher in Tabata-HIIT compared to SSG (Z = −4.284, *p* < 0.001, r = 0.760).

**Figure 7:**
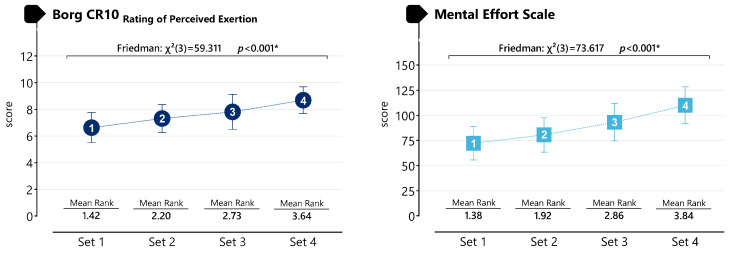
Perceived levels of strain and mental effort during SSG. * *p* < 0.05.

The results presented in Figure 7 indicated statistically significant differences across sets for perceived exertion (Borg CR-10) and mental effort during the SSG condition. Friedman analysis revealed a statistically significant effect of set number on perceived exertion (χ^2^(3) = 59.311, *p* < 0.001) and mental effort (χ^2^(3) = 73.617, *p* < 0.001), demonstrating that ratings differed significantly across sets.

## 4. Discussion

This study compared the physiological, neuromuscular, perceptual, and cognitive responses to SSG and Tabata-style HIIT in order to examine the acute effects of different high-intensity training methods applied to amateur soccer players. The findings revealed that while both exercise approaches imposed a similarly high load on the cardiovascular system, they elicited distinct response patterns in terms of neuromuscular performance, perceived exertion, mental effort levels, and short-term recovery processes. These findings demonstrate that the acute responses observed during high-intensity exercise are not solely attributable to the magnitude of the physiological load; rather, the type of exercise, its delivery method, organization, and task-specific characteristics also play a significant role in the development of these responses [20].

### 4.1. Physical Performance and Acute Neuromuscular Responses

The results of this study indicate a significant decline in CMJ performance following Tabata-style HIIT. However, no similar change in performance was observed among participants following the SSG protocol. Although there was a tendency toward a decrease in SJ performance values following the Tabata-HIIT protocol, the difference between the two training methods was found to be statistically insignificant. These results are consistent with the findings of previous studies indicating that HIIT with short recovery intervals can induce acute neuromuscular fatigue in athletes, which may lead to temporary performance declines in explosive force production [40,41]. The metabolic and physiological stress pattern created by the Tabata protocol could be a contributing factor to the observed decline in performance. In this training method, while high levels of glycolytic energy production are activated, it could lead to the rapid depletion of phosphocreatine stores. Additionally, the short rest periods used may not provide sufficient time for the regeneration of this energy system. It is believed that these physiological conditions may be one of the factors explaining the decline in jump performance [42].

Analyses of sprint and agility performance showed that no statistically significant changes occurred following either exercise protocol. This finding suggests that acute neuromuscular fatigue caused by high-intensity exercise may not necessarily have a pronounced effect on linear sprint speed or the ability to change direction. Furthermore, considering the total duration and structural characteristics of the training protocols applied, it is also plausible that the loads in question were not of a magnitude sufficient to cause a measurable decline in sprint and agility performance.

SSG present a dynamic loading model characterized by intermittent play sequences and various movement patterns, despite placing high physiological demands on participants. These characteristics may contribute to the distribution of mechanical and metabolic load across a broader range of movements and a relatively more balanced distribution of load among muscle groups during exercise [39,43]. Findings in the literature also support this approach. For example, Selmi et al. [37] reported that 4-on-4 SSG and running-based HIIT protocols elicited similar responses in terms of cardiovascular and metabolic stress, but lower-limb muscle strength was maintained at a higher level following SSG. The results obtained in this study are also consistent with these findings. Indeed, no significant decrease in neuromuscular performance was observed following the SSG session. This suggests that game-based training formats may create a different load distribution compared to linear running-based high-intensity workouts, potentially limiting acute performance losses.

### 4.2. Cardiovascular Responses and Perceived Strain

Training load is commonly quantified using both physiological and perceptual markers, including RPE, heart rate (HR), and blood lactate concentration, with HR serving as one of the most widely applied objective indicators of exercise intensity [44].

Analysis of physiological data related to HR revealed that SSG and Tabata-style HIIT induced significant cardiovascular stress in participants. The fact that the HR_mean_, HR_peak_, and HR_max_ percentages were at similar levels indicates that both types of exercise were performed at comparable levels.

The similarity in HR values recorded during different sets of the SSG session indicates that exercise intensity remained largely constant throughout the duration of the session. It has been demonstrated that SSG can generate high-intensity physiological loads capable of reaching approximately 85–95% of athletes’ HR_max_ [43]. This indicates that SSG formats can be considered an effective training method capable of providing a sustainable and consistent cardiovascular load throughout a training session.

Although both training protocols impose a similar level of cardiovascular load, different trends were observed in terms of athletes’ subjective perceptions and mental responses. In particular, the fact that perceived exertion and mental effort levels remain higher during the recovery period following the Tabata-HIIT protocol suggests that this training model may impose a more pronounced and longer-lasting perceptual load on athletes. This finding demonstrates that responses to exercise may not be fully explained solely by physiological intensity; the manner in which exercise is performed and its structural characteristics also play a significant role in athletes’ processes of perceiving and experiencing exercise. Indeed, Selmi et al. [45], in their study comparing HIIT and SSG, reported that while both methods elicited similar physiological responses, HIIT sessions were associated with more negative emotional states and higher perceived exertion, whereas the SSG format provided athletes with a more balanced and tolerable exercise experience.

Previous studies have shown that training formats can be perceived differently by athletes, even when exercise intensity is similar. In this context, Selmi et al. [37] reported that SSG were rated by athletes as a more enjoyable and tolerable exercise experience compared to HIIT protocols. The findings of this study are consistent with these results. Indeed, it was determined that perceived exertion and mental effort levels remained higher following the Tabata-style HIIT session, and the decrease in these variables during the recovery period was more limited. This suggests that the Tabata-HIIT protocol may impose a more pronounced subjective and cognitive load on participants.

Studies conducted across various sports disciplines have demonstrated that this distinction between perceptual and physiological responses is not unique to the context of soccer. In this regard, Simonelli et al. [46], in a study comparing HIIT, SSG, and full-field games among elite handball players, reported that while HIIT and SSG training produced similar levels of cardiovascular load, significant differences emerged in terms of the athletes’ subjective responses. According to the study’s findings, HIIT sessions were associated with higher perceived physical exertion, mental load, and stress responses, whereas SSG were evaluated by athletes as a more manageable and balanced exercise experience. These results support the high levels of perceived exertion and mental effort observed following Tabata-style HIIT in the present study. Therefore, not only the physiological intensity of exercise but also its form of implementation and structural characteristics are among the key factors shaping athletes’ perceptual experience. In this study, HR-based indicators were used to assess exercise intensity. However, it is known that exercise load is not limited to cardiovascular responses alone but exhibits a multidimensional structure that includes metabolic, neuromuscular, and mechanical components [20,47]. Therefore, it should be considered that similar HR responses may be associated with different internal and external load profiles. In this context, the absence of statistically significant differences in HR-based parameters between protocols should not be interpreted as indicating that these exercise models are physiologically equivalent. The current findings merely demonstrate that cardiovascular responses are similar and do not allow for direct inferences regarding other components of exercise load.

### 4.3. Mental Effort and Training Tolerance

Research findings indicate that athletes’ mental effort levels increased gradually as sets progressed during SSG. In contrast, it was observed that mental effort levels remained high during the recovery period following Tabata-style HIIT. It has been reported that mental fatigue can negatively affect physical performance and increase the perceived level of exertion [48]. In this context, it is thought that the intense physiological and cognitive load induced by the Tabata-HIIT protocol over a short period of time may have led athletes to require a longer rest period.

The study conducted by Xu et al. [49] also points to a similar trend. In this study conducted on young adult soccer players, it was reported that SSG and HIIT protocols produced similar physiological loads in terms of exercise intensity. However, it was reported that SSG, particularly in 1-on-1 and 5-on-5 formats, yielded higher scores on the Physical Activity Enjoyment Scale (PACES). This finding suggests that game-based training may be perceived as more enjoyable by athletes and could increase their tolerance for exercise. Therefore, it can be argued that SSG may offer a training approach that not only creates a physiological load but also supports athletes’ participation in training and its long-term sustainability.

### 4.4. Cognitive Performance

The results of the d2 attention test administered following the exercise protocols indicated significant changes in the participants’ cognitive performance. Post-exercise assessments revealed marked increases in TN and CP scores, while a significant decrease was observed in Total Errors scores. However, the absence of a statistically significant difference between the SSG and Tabata-HIIT protocols suggests that high-intensity exercise may acutely improve attention, concentration, and overall cognitive performance regardless of the exercise format [22,23].

The current literature also indicates that even a single exercise session can produce short-term improvements in cognitive function. In particular, it has been reported that acute high-intensity exercise can lead to temporary changes in neurophysiological processes and mechanisms related to executive functions [25,50]. However, the number of studies directly examining the effects of soccer-specific training approaches on cognitive performance remains limited. In a study conducted with children, Lind et al. [51] reported that high-intensity 3-on-3 SSG led to improvements in neurophysiological indicators associated with attention and inhibitory control. Similarly, Hammami et al. [52] reported in their study of adolescent athletes that both SSG and sprint-based exercise acutely improved attention performance as assessed by the d2 attention test.

Although Birinci et al. [53] did not detect a significant change in serum BDNF levels, they reported that narrow-field games (SSG) produced more pronounced improvements in cognitive processing speed compared to running-based HIIT. These findings demonstrate that acute changes in cognitive development may not be fully explained solely by neurochemical changes. Furthermore, they suggest that the perceptual complexity and task-oriented demands inherent in exercise may also contribute to the formation of mental responses.

It should be noted that the cognitive benefits associated with sports activities may not manifest similarly across all cognitive tests, and that sudden changes in performance may be influenced by the characteristics of the assessment tools used [54]. Furthermore, soccer’s unique game dynamics require athletes to continuously monitor information in their surroundings during play, respond rapidly to changing stimuli, and make quick decisions under time pressure. On the other hand, the ecological dynamics approach examines this process from the perspective of the interaction between perception and action, emphasizing that context-dependent decision-making processes operate more intensively, particularly in close-range play [26].

From an ecological dynamics perspective, performance in team sports emerges from the continuous interaction between the individual, task, and environment, where perception and action are tightly coupled processes [55,56]. In SSG, these interactions are intensified due to reduced space, time pressure, and constantly changing constraints, which may increase demands on attention, information processing, and decision-making. In this context, the cognitive responses observed in the present study may be partly associated with these task-specific demands rather than being solely attributed to physiological factors. Previous research also suggests that perception–action coupling and representative learning environments can contribute to the development of perceptual–cognitive skills in sport [57]. However, given the absence of direct neurophysiological or process-based measurements in the present study, these interpretations should be considered as plausible explanations rather than definitive mechanisms.

The timing of cognitive assessments should also be considered when interpreting the current findings. Cognitive responses to acute exercise are time-dependent, and different results can be obtained when performance is assessed immediately after exercise compared to when assessed after a short recovery period [22,23]. In this study, cognitive measurements were taken immediately after the exercise protocols to capture acute responses, while baseline assessments were performed before warming up to minimize potential fatigue-related effects. Therefore, the observed changes in cognitive performance may reflect transient post-exercise responses rather than more stable cognitive adaptations.

When interpreting the increases in cognitive performance observed in this study, the learning effect associated with repeated test administration must not be overlooked. The literature indicates that administering the same cognitive test at short intervals may lead to significant increases in performance, and that these increases may reflect the test–retest effect rather than actual cognitive development [58,59]. Therefore, interpreting the cognitive changes observed in the present study solely as acute effects of exercise provides a limited interpretation.

### 4.5. Practical Meaning and General Evaluation

To prevent the accumulation of fatigue, it is recommended to incorporate Tabata-style HIIT sessions into the training program, ensuring sufficient rest periods are included. On the other hand, on days when cognitive and perceptual demands are particularly high, SSG can be considered a suitable training option. While studies show that SSG and Tabata-HIIT provide similar cardiovascular loads, they reveal that the Tabata-HIIT protocol is associated with greater perceived exertion, increased mental load, and limited rest periods among athletes. On the other hand, it has been determined that SSG help maintain high physiological stimulation while also preserving neuromuscular performance and providing a more balanced perceptual load for athletes. These findings emphasize the importance of considering not only physiological load but also perceptual, mental, and neuromuscular responses when conducting high-intensity training. HIIT is reported to offer broader physiological benefits beyond just athletic performance; it may be associated with improvements in parameters such as bone remodeling, lipid profile, and physical function [60]. It should be noted that the training protocols being compared have structurally distinct characteristics when interpreting the findings. In this context, it is believed that the observed responses may not be explained solely by exercise intensity; factors such as the organizational structure of the protocols, movement variety, and task demands may also play a role in these responses. While the cross-over design offers significant advantages in reducing between-subject variability and ensuring that each participant experiences all conditions, the potential effects of order and carryover effects on the results should not be overlooked [61]. The fact that these effects were not directly analyzed statistically in this study should be considered a methodological limitation that must be taken into account when interpreting the findings.

From a practical perspective, the findings of this study demonstrate that high-intensity training formats can be flexibly integrated into training programs, depending on the specific context and training goals. The observed responses show that such training approaches can be used to create meaningful physiological and perceptual demands while also engaging cognitive processes. However, these applications should be carefully considered, taking into account the specific characteristics of the athletes and training conditions. Coaches can also consider the timing and sequence of training sessions within the weekly periodization to balance physical and cognitive demands.

### 4.6. Limitations

Several limitations should be considered when evaluating the findings of this study. First, the fact that the participants consisted only of amateur male soccer players limits the direct generalization of the results to female athletes, different age groups, or professional-level athletes. Given that responses to exercise can vary in individuals with different performance levels, training histories, and physiological characteristics, generalizing these findings to broader athlete populations should be approached with caution. Additionally, since the study focused solely on the immediate, or short-term, effects of exercise, it is not possible to make definitive statements about changes that long-term training might produce. Cognitive performance was assessed solely using the d2 attention test, which did not allow for a broader examination of executive functions. Furthermore, administering the same test at short intervals increases the likelihood of a practice effect. Additionally, the absence of a control condition without exercise limits the ability to attribute the observed cognitive changes directly to exercise. Using parallel test forms or designs that include a control group in such cognitive assessments could yield stronger results [59].

Although a cross-over design was used in the study, potential order and carryover effects related to the protocol sequence were not directly analyzed. Additionally, while the 48-h recovery period between protocols was chosen to reduce acute fatigue, it does not guarantee that residual effects, particularly those in the neuromuscular and cognitive systems, have completely dissipated [28].

Another limitation of the study is that potentially influencing variables such as nutrition, hydration, sleep quality, and previous training load were not objectively monitored or controlled for, and this may have affected both physical and cognitive outcomes.

The comparison of training load between protocols is primarily based on HR responses, which may not fully reflect differences in external or mechanical load. The absence of additional measurements, such as GPS data or other internal load indicators, should be considered when interpreting the findings.

## 5. Conclusions

The study results indicate that the two training programs examined provided similar levels of cardiovascular load, and suggest that HIIT protocols, specifically the SSG protocol and Tabata-style training, may be associated with differences in acute neuromuscular and perceptual responses in amateur soccer players. It was observed that the Tabata-HIIT method is associated with a temporary decline in neuromuscular performance, along with greater perceived exertion and increased mental strain. On the other hand, small-group games were found to help maintain neuromuscular performance under similar physiological load conditions and to offer a more balanced perceptual load profile for athletes. Additionally, it was found that both training methods similarly provide immediate improvements in attention and CP, and there is no significant difference in cognitive outcomes between these protocols. These findings emphasize the importance of considering not only physical load but also perceptual, cognitive, and neuromuscular responses when planning soccer training. The fact that HR-based responses are similar in both training models may not directly indicate complete physiological equivalence across all components of exercise load. It should be noted that the observed improvements in cognitive performance may stem not only from acute exercise-related effects but also from potential test–retest effects.

## Figures and Tables

**Table 1:** Descriptive Characteristics of Participants (*n* = 32).

Variable	Mean ± SD	Minimum	Maximum
Age (years)	20.53 ± 1.65	18	24
Height (cm)	176.41 ± 3.83	170.00	187.00
Body Mass (kg)	69.47 ± 7.34	57.40	83.20
Skeletal Muscle Mass (kg)	33.53 ± 3.63	20.70	40.10
Body Fat (%)	15.14 ± 4.87	6.70	24.50
Training Experience (years)	6.53 ± 1.81	5.00	11.00

## Data Availability

The data supporting the findings of this study are available from the corresponding author upon reasonable request.

## Supplementary Materials

The following supporting information can be downloaded at: https://www.mdpi.com/article/10.3390/life16040646/s1, Supplementary Table S1: Summary Table of Main Results.

## Abbreviations

%HR_max_	Percentage of maximal heart rate
%HR_mean_	Percentage of mean heart rate
ANOVA	Analysis of variance
CMJ	Countermovement jump
CP	Concentration performance
HIIT	High-intensity interval training
HR	Heart rate
HR_mean_	Mean heart rate
HR_peak_	Peak heart rate
RPE	Rating of perceived exertion
SD	Standard deviation
SJ	Squat jump
SSG	Small-sided games
TN	Total number of items processed
