# Supplementary material for: Acute Effects of Small-Sided Games and Tabata High-Intensity Interval Training on Physical, Psychophysiological, and Cognitive Responses in Male Soccer Players
Source: Life (Basel). 2026 Apr 11;16(4):646. doi: 10.3390/life16040646 (PMC13117200; doi:10.3390/life16040646)
Supplement: Supplementary file 1 [file life-16-00646-s001.zip › life-4239112-supplementary.pdf]

Supplementary Table S1. Summary Table of Main Results

Variable	Protocol	Pre (Mean ± SD)	Post (Mean ± SD)	Δ (Mean ±SD)	p	Cohen's d	95% CI LB	95% CI UB	F (1,31)	p	η^2p
CMJ (cm)	SSG	36.78 ± 4.41	37.53 ± 5.50	0.75± 3.50	0.237	0.21	-2.010	0.516	18.20	<0.001**	0.370
	Tabata-HIIT	36.80 ± 4.92	34.45 ± 5.67	-2.35 ±3.39	< 0.001**	0.69	1.126	3.568			
SJ (cm)	SSG	35.97 ± 4.94	35.82 ± 5.89	-0.15± 2.26	0.716	0.07	-0.669	0.963	3.49	0.071	0.100
	Tabata-HIIT	35.42 ± 5.01	34.15 ± 5.55	-1.27± 2.75	0.014*	0.46	0.273	2.258			
20 Meter Sprint (s)	SSG	3.20 ± 0.20	3.24 ± 0.18	0.04 ± 0.18	0.246	0.21	-0.102	0.027	0.11	0.747	0.003
	Tabata-HIIT	3.16 ± 0.18	3.21 ± 0.16	0.05± 0.17	0.109	0.29	-0.108	0.011			
Agility (s)	SSG	10.66 ± 0.71	10.64 ± 0.64	0.02± 0.32	0.741	0.06	-0.098	0.136	0.03	0.871	0.001
	Tabata-HIIT	10.71 ± 0.58	10.67 ± 0.62	0.03± 0.31	0.563	0.10	-0.080	0.144			
TN	SSG	192.09 ± 48.75	234.28 ± 42.53	42.19 ± 25.63	< 0.001**	1.65	-51.43	-32.95	0.04	0.842	0.001
	Tabata-HIIT	204.37 ± 50.84	248.03 ± 45.74	43.66 ± 26.65	< 0.001**	1.64	-53.27	-34.05			
Total Error	SSG	472.22 ± 51.13	433.19 ± 44.49	-39.03± 25.60	< 0.001**	1.52	29.80	48.26	0.29	0.595	0.009
	Tabata-HIIT	461.63 ± 51.66	418.69 ± 47.67	-42.94± 26.30	< 0.001**	1.63	33.46	52.42			
CP	SSG	185.88 ± 51.07	224.47 ± 44.24	38.59± 25.24	< 0.001**	1.53	-47.69	-29.49	0.26	0.614	0.008
	Tabata-HIIT	197.13 ± 51.29	239.38 ± 47.74	42.25± 26.24	< 0.001**	1.61	-51.71	-32.79			

Variable	SSG (Mean ± SD)	Tabata-HIIT (Mean ± SD)	Δ (%)	p	Cohen's d	95% CI LB	95% CI UB
HR_{mean} (bpm)	180.08 ± 7.67	178.99 ± 9.14	1.08	0.425	0.14	-0.206	0.490
HR_{peak} (bpm)	191.19 ± 6.25	190.05 ± 7.10	1.13	0.102	0.30	-0.059	0.650
$\%HR_{mean}$	90.27 ± 3.65	89.72 ± 4.50	0.55	0.418	0.15	-0.063	0.645
$\%HR_{max}$	95.84 ± 2.88	95.28 ± 3.52	0.56	0.107	0.29	-0.204	0.492

Variable	Protocol	Measurement Time	Mean ± SD	Z	p	Effect size (r)	95% CI LB	95% CI UB
Borg CR10	SSG	End of Set	8.69 ± 1.00	-4.284	<0.001**	0.760	0.816	0.965
		10 min Post	7.19 ± 1.23					
	Tabata-HIIT	End of Set	9.31 ± 0.82	-2.422	0.015*	0.430	0.192	0.824
		10 min Post	8.66 ± 1.47					
	SSG vs	10 min Post	7.19 ± 1.23	-3.499	<0.001**	0.620	-0.901	-0.537
	Tabata-HIIT	10 min Post	8.66 ± 1.47					
Mental Effort Scale	SSG	End of Set	110.00 ± 18.32	-4.376	<0.001**	0.774	0.838	0.967
		10 min Post	83.13 ± 26.33					
	Tabata-HIIT	End of Set	117.19 ± 19.55	-2.762	0.006*	0.490	0.328	0.866
		10 min Post	108.44 ± 22.16					
	SSG vs	10 min Post	83.13 ± 26.33	-4.284	<0.001**	0.760	-0.943	-0.744
	Tabata-HIIT	10 min Post	108.44 ± 22.16					

* $p < 0.05$; ** $p < 0.001$; SD, standard deviation; Δ, percentage change; CI, confidence interval; LB, lower bound; UB, upper bound; η^2p, partial eta squared; SSG, small-sided games; HIIT, high-intensity interval training; CMJ, countermovement jump; SJ, squat jump; TN, total number of items processed; CP, concentration performance; HR_{mean}, mean heart rate; HR_{peak}, peak heart rate; $\%HR_{mean}$, percentage of mean heart rate; $\%HR_{max}$, percentage of maximal heart rate
